# Self-assembled micelles of amphiphilic PEGylated rapamycin for loading paclitaxel and resisting multidrug resistant cancer cells†
†Electronic supplementary information (ESI) available: Chemicals and reagents, detailed experimental procedures for materials synthesis, characterization, cellular evaluations and supporting figures and tables. See DOI: 10.1039/c4tb01633e



**DOI:** 10.1039/c4tb01633e

**Published:** 2015-01-27

**Authors:** Wei Tian, Jieying Liu, Yuan Guo, Yuanyuan Shen, Dejian Zhou, Shengrong Guo

**Affiliations:** a School of Pharmacy , Shanghai Jiao Tong University , Shanghai , 200240 , China; b School of Chemistry and Asbury Centre for Structural Molecular Biology , University of Leeds , Leeds , LS2 9JT , UK . Email: d.zhou@leeds.ac.uk ; Email: s.guo@leeds.ac.uk ; Email: srguo@sjtu.edu.cn ; Tel: +44 (0)113 3436230 ; Tel: +44 (0)113 3436449

## Abstract

Self-assembled micelles of amphiphilic PEG–rapamycin conjugates loaded with paclitaxel have been developed for co-delivery and simultaneous intracellular release of paclitaxel and rapamycin, bypassing the cancer cell drug resistant mechanism and maximising the synergy of dual-drug combinational therapy. This novel nanomedicine offers 20-fold improved potency over free paclitaxel against a model multidrug resistant human breast cancer cell.

## Abstract 2

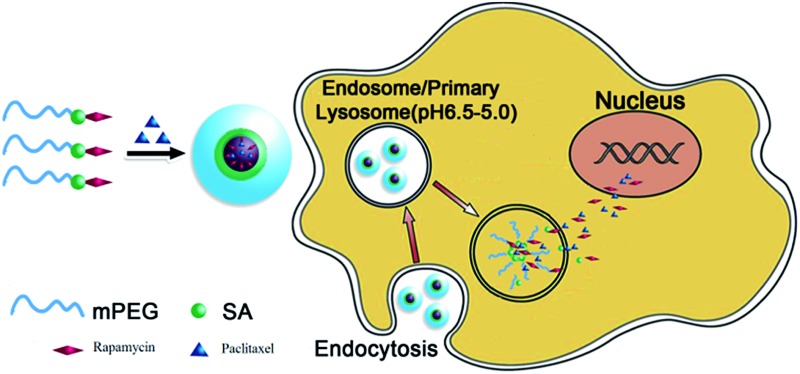
A paclitaxel loaded PEGylated rapamycin micelle effectively bypasses the resistance mechanism, allowing for potent treatment of multidrug resistant cancer cells.

Multidrug resistance (MDR) is a leading cause of chemotherapy failure in cancer treatment. Overcoming MDR is a tremendous challenge faced by the pharmaceutical and healthcare industries currently.^1^ Over-expressed efflux transporter proteins (*e.g.* g-glycoprotein, MDR1 and MDR associated proteins) are widely found on MDR cells which can effectively remove drugs from the cell interior, preventing drug accumulation and compromising treatment efficacy. To address this challenge, one strategy has been the co-delivery of anti-cancer drugs and specific inhibitors against such efflux transporters to increase the drug accumulation and improve treatment efficacy.^2^ Another strategy has been the development of nanoscale drug formulations (*i.e.* nanomedicines) which have completely different cell entry mechanisms from free drugs (*e.g.* endocytosis *vs.* diffusion) and can deliver their drug payloads deep into the cell interior.^3^ As a result, nanomedicines can bypass the drug resistance mechanisms of MDR cells, leading to improved treatment efficacy. Furthermore, two or more different drugs can also be combined to exploit the synergy of multi-drug treatment.^3^ Despite significant research, the full potentials of synergistic treatment is rather difficult to realise because of the different drug release kinetics, making simultaneous co-delivery and release of multiple drugs in target cells difficult. To address this challenge, herein we have proposed a new approach where a PEGylated amphiphilic drug molecule micelle is directly employed as the carrier for a second hydrophobic drug, leading to convenient loading and simultaneous intracellular release of two different drugs (Scheme 1) to maximise the synergy of dual-drug treatment. We show that these novel dual-drug loaded micelles are highly effective against the MCF-7/ADR cell line, a MDR human breast cancer cell model.

**Scheme 1:**
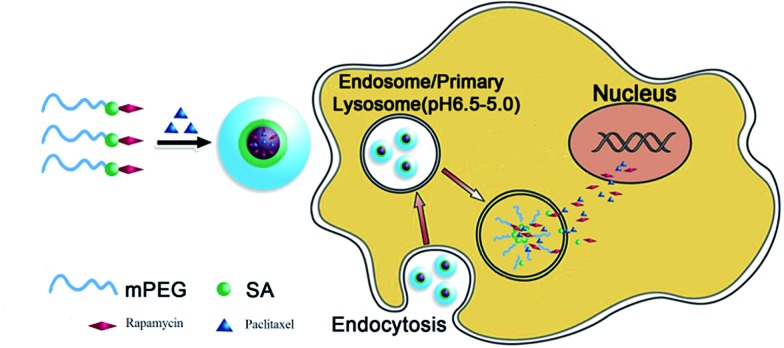
Schematics of the fabrication and intracellular drug release of PTX-loaded mPEG–SA–rapamycin micelles. Rapamycin is conjugated to PEG *via* an acid-liable ester bond to obtain amphiphilic mPEG–SA–rapamycin which then self-assemble into a micelle in the presence of paclitaxel, leading to simultaneous paclitaxel loading in the core. After cell uptake, hydrolysis of the ester bonds between PEG and rapamycin under the acidic intracellular compartments (*e.g.* endosome/lysosome) leads to the micelle disassembly, triggering the simultaneous release of both paclitaxel and rapamycin for synergistic dual-drug therapy.

Scheme 1 shows the schematics of our approach and the proposed working mechanism. Firstly, hydrophobic rapamycin is PEGylated *via* an ester bond formation to yield mPEG–SA–rapamycin, turning rapamycin amphiphilic.^5^ mPEG–SA–rapamycin is then exploited for micelle assembly with paclitaxel, a second potent hydrophobic anticancer drug widely used in clinical treatment of breast, ovarian, colon, bladder, lung, and head and neck cancers,^6^ yielding paclitaxel loaded rapamycin–PEG micelles (Scheme 1, left). The loading of paclitaxel is presumably through hydrophobic van der Waals interactions between the hydrophobic paclitaxel and paramycin moieties, forming dual-drug loaded hydrophobic micelle cores. After uptake, the micelles are internalised into endosomes, which are gradually acidified following the natural endosomal maturation process, triggering the cleavage of the acid-labile ester linkage between the PEG and rapamycin and the break up of the micelles. As a result, paclitaxel and rapamycin are released simultaneously inside the intracellular compartments, which can then diffuse across the endo-/lyso-somal membranes into cytosol and/or nucleus to exert their therapeutic functions. The simultaneous intracellular release of both drug loads provides an ideal situation for maximising the synergy of dual-drug treatment.

PEGylated rapamycin is chosen as the model amphiphilic drug carrier here because it is a hydrophobic macrolide with useful immune suppressing functions. It has been used clinically to reduce rejection in organ transplantation and also used as a coronary stent coating. Recently, rapamycin was found to have broad activities against lung, cervix, colon and breast cancers.^4^ It has also been combined with paclitaxel to exploit combinational therapy. For example, Shafer *et al.* found that rapamycin potentiated the effects of paclitaxel in endometrial cancer cells through inhibition of cell proliferation and induction of apoptosis, and potentially increased polymerization and acetylation of tubulin. Their results suggest that the combined rapamycin–paclitaxel treatment may be beneficial for endometrial cancer treatment.^7^ Moreover, Mishra *et al.* found that paclitaxel–rapamycin dual-drug loaded poly(ethylene glycol)-*block*-poly(d,l-lactic acid) (PEG-*b*-PLA) micelles had good anti-angiogenic effects at the cellular level.^8^ However, such studies were either based on the physical mixture or co-encapsulated paclitaxel–rapamycin, it is difficult to achieve co-delivery and simultaneously release both drugs inside target cells to maximise their synergy.

mPEG–SA–rapamycin was prepared by a standard dicyclohexylcarbodiimide/dimethylaminopyridine based ester coupling chemistry. Briefly, mono-methoxyl PEG (MW = 2000) was reacted with succinic anhydride to yield methoxyl-poly(ethylene glycol)–succinic acid (mPEG–SA), which was then coupled to rapamycin *via* esterification to form mPEG–SA–rapamycin (see Scheme S2, ESI† for details). Each mPEG–SA–rapamycin molecule contains a hydrophilic PEG chain and a hydrophobic rapamycin moiety as confirmed by its NMR spectrum and TLC analysis (Fig. S1, ESI†). The equivalent rapamycin weight loading in mPEG–SA–rapamycin is ∼30%. mPEG–SA–rapamycin is highly soluble in water, 16.7 mg mL^–1^ (or 5.0 mg mL^–1^ rapamycin equivalent), which is ∼1900 fold higher than free rapamycin (2.6 μg mL^–1^). PEGylation is one of the most effective and widely used approaches to improve solubility and bioavailability of hydrophobic drugs.^5^ Besides, the amphiphilic nature of mPEG–SA–rapamycin makes it easily self-assemble into uniform, nanosized micelles in water with a hydrodynamic diameter of 56 ± 3 nm (polydispersity index (PDI) = 0.132, see ESI, Fig. S1†). It has a low critical micelle concentration (CMC) of only 11.4 μg mL^–1^.

The mPEG–SA–rapamycin micelles can be used as a carrier for other hydrophobic drugs, providing a unique opportunity for exploiting the synergy of dual-/multi-drug therapy. Here paclitaxel was selected as the second anticancer drug because it is hydrophobic and highly effective against a wide range of human cancers. Paclitaxel was encapsulated into the mPEG–SA–rapamycin micelles with a high loading efficiency (93.3 ± 1.7%) by a simple solid dispersion method without any additional surfactants or additives (see ESI† for details). Encapsulation of hydrophobic paclitaxel into the hydrophobic core led to a considerable increase of micelle hydrodynamic size from ∼56 to ∼94 nm, but retained a similar size uniformity (PDI = 0.175). Importantly, paclitaxel encapsulation was found to improve the micelle stability significantly: the paclitaxel-loaded micelles can be lyophilized and then reconstituted into clear aqueous solutions without observable change of hydrodynamic size, presumably because the strong attractive hydrophobic interactions between the encapsulated paclitaxel and rapamycin greatly stabilise the micelle cores. It also retained a very similar zeta potential (–11.2 ± 1.2 mV) as the parent micelle (10.6 ± 2.0 mV). The weight loading of paclitaxel in the micelle was 8.4 ± 0.1%, while the equivalent rapamycin loading was 27.5%, benefited from the micelles made of the PEGylated drug molecules.

Nanomedicines are mostly taken up by cells *via* endocytosis, and hence will be exposed to a gradually increasing acidic environment as a result of the natural endosomal maturation process. Here the *in vitro* drug release profiles were studied at pH 7.4 and 5.0. The release profiles for both rapamycin and paclitaxel were pH sensitive: rapid releases were found at pH 5.0 (similar to that of late endosome or lysosome) where ∼90% of the drugs were released in the first 10 h. At pH 7.4 (normal physiological pH), the drug release was much slower, where maximum release was not reached until after 72 h (Fig. 1). The rapid release observed at pH 5.0 is likely due to the effective cleavage of the acid-labile ester linkage between the rapamycin and PEG under such pH, leading to micelle destruction and hence release of both drugs. The fact that both drugs are rapidly and simultaneously released at pH 5.0 makes our micelle system well-suited for intracellular co-delivery of paclitaxel/rapamycin by exploiting the gradual acidification of the natural endosomal trafficking/maturation process, which should benefit and maximise the synergy of dual-drug combinational treatment.

**Fig. 1:**
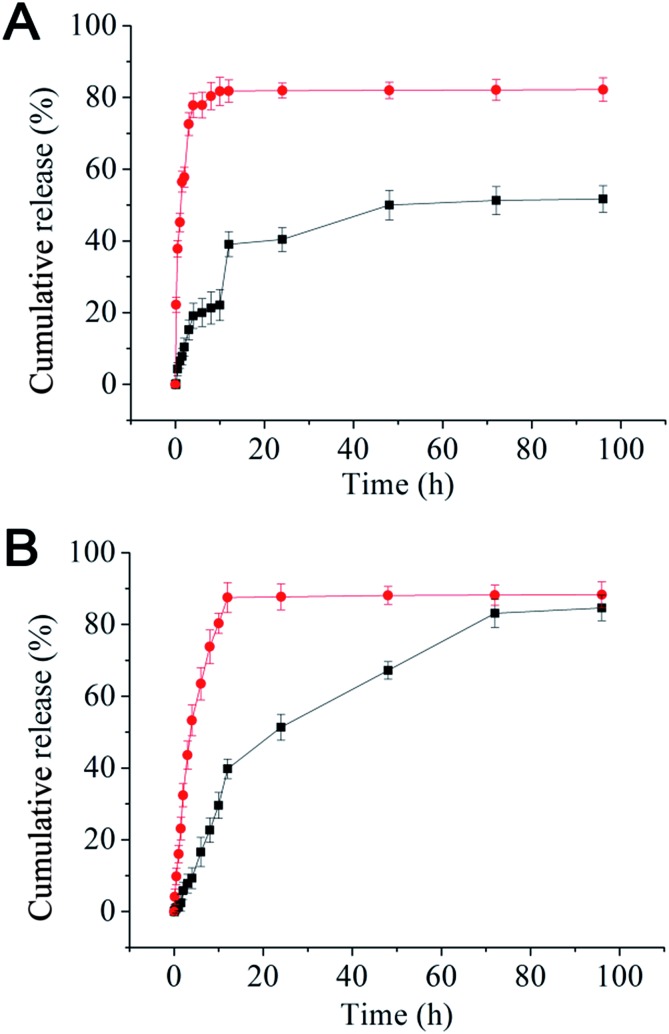
Release profiles for rapamycin (A) and paclitaxel (B) from the mPEG–SA–rapamycin micelles at pH 5.0 (red dots) and 7.4 (black squares) in PBS containing 1% Tween-80 at 37 °C. Data are given as mean ± SD (*n* = 3).

Effective removal of therapeutics from the cell interior by over-expressed efflux transporters is a major cause of MDR in cancer cells. Here we have evaluated the uptake and efflux of the mPEG–SA–rapamycin micelles encapsulated with Rhodamine 6G (R6G) on the human breast cancer cells, MCF-7 (drug-sensitive) and MCF-7/ADR (drug-resistant) by monitoring the cellular R6G fluorescence after treatment *via* flow cytometry. R6G is a suitable fluorescence probe because it is a substrate for p-glycoprotein, a major efflux transporter of MDR cells. Fig. 2A shows that the R6G fluorescence in MCF-7 cells is much stronger than that of the MCF-7/ADR cells throughout the 24 h incubation period with free R6G, indicating much less accumulation of R6Gs in the MCF-7/ADR cells over the MCF-7 cells. This agrees well with that the over-expressed *p*-glycoproteins on the MDR cell surface can effectively efflux internalised R6Gs, preventing its intracellular accumulation. At 24 h, the R6G intensity ratio between the drug-sensitive and -resistant cells was ∼106 fold with free R6G treatment (530 *vs.* 5). This ratio was decreased to ∼9 fold with the R6G-micelle treatment (630 *vs.* 70), suggesting a dramatically decreased efflux ability for the MCF-7/ADR cells against the R6G-micelles. Moreover, the micelle treated MCF-7/ADR cells yielded a R6G fluorescence intensity 14 times as strong as those treated by free R6G, suggesting that the micelles may have good potential for overcoming drug resistance in the MCF-7/ADR cells. Consistent with this observation, the R6G efflux rate for the R6G-micelle treated MCF-7/ADR cells was also significantly lower than those treated with free R6G (Fig. 2B), presumably because the micelle encapsulation completely altered the cell entry pathway for R6G (endocytosis rather than diffusion), allowing the R6G payload to be delivered and released deeply into the cell interior, significantly reducing the efficiency of efflux transporters located on the MCF-7/ADR cell membranes.

**Fig. 2:**
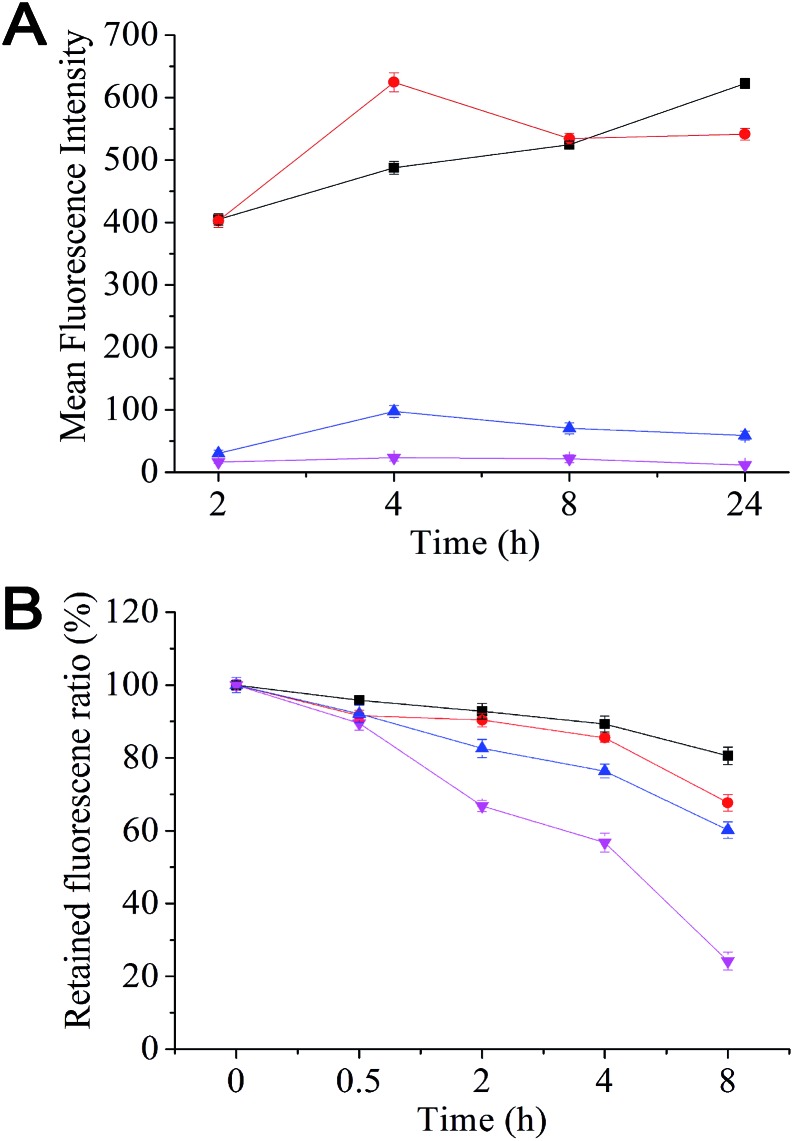
(A) Uptake and (B) efflux of Rhodamine 6G-loaded mPEG–SA–rapamycin micelles and free Rhodamine 6G by MCF/7 (black squares and red dots) and MCF-7/ADR cells (blue triangles and pink inverted triangles).

The antitumor mechanism of rapamycin is to bind FKBP12 and inhibit mTOR, a central cell growth and proliferation regulator. mTOR inhibition results in cell-cycle arrest in the G1 phase. In addition, rapamycin also blocks NF-nB activation and may sensitize drug-resistant cells to chemotherapeutic treatment. Quantitative analysis of the cell cycle distribution revealed that only 56.79% of the untreated cells were in the G0/G1 phase, which was increased to 69.42% and 68.14% after treatments with rapamycin and mPEG–SA–rapamycin respectively (see ESI, Table S1†). This suggests that both rapamycin and mPEG–SA–rapamycin have the same antitumor mechanism, *i.e.* blocking cell cycle progression from the G1 to S phase. Hence the antitumor effect of mPEG–SA–rapamycin may originate from free rapamycin after hydrolysis, which is in good agreement with the proposed action mechanism shown in Scheme 1.

Both rapamycin and mPEG–SA–rapamycin inhibited the MCF-7 cell growth in a dose-dependent manner (ESI, Fig. S3†). The IC_50_ values of rapamycin and mPEG–SA–rapamycin against the MCF-7 cells were found to be 0.20 and 1.02 μg mL^–1^ (for rapamycin or equivalent), respectively. The IC_50_ value of mPEG–SA–rapamycin against MCF-7 cells is comparable to that of a PEGylated rapamycin against the CRL 1739 human gastric adenocarcinoma cell lines (1 μg mL^–1^) recently reported by Kumar and Lokesh.^9^ This indicates that PEGylated rapamycin has broad antitumor activities against different types of cancer cells. The cytotoxicity of mPEG–SA–rapamycin against MCF-7 cells was lower than that of free rapamycin. However, their cytotoxicity results against the MCF-7/ADR cells were exactly the opposite: where mPEG–SA–rapamycin was more cytotoxic than free rapamycin (IC_50_ values: 3.48 *vs.* 10.50 μg mL^–1^, see ESI Table S2 and Fig. S4†). This result is consistent with the earlier observation that the MCF-7/ADR cells are less effective at effluxing the R6G–mPEG–SA–rapamycin micelles than free R6G. The resistance indexes of rapamycin and mPEG–SA–rapamycin were 52.5 and 3.4, respectively, indicating that PEGylation of rapamycin significantly reduced the resistance of the MCF-7/ADR cancer cells.

The IC_50_ values of the different paclitaxel formulations against the MCF-7 and MCF-7/ADR cells are summarised in Table 1. Compared to the MCF-7 cells, the MDR MCF-7/ADR cells are highly resistant to free paclitaxel treatment with a high resistance index of 212, which is much higher than that against rapamycin. Interestingly, a physically combined paclitaxel and rapamycin treatment (1 : 3 w/w, similar to that of the drug ratio in the paclitaxel loaded micelles) exhibited a good antitumor synergy against the MCF-7 cells, with a 5.1 fold reduction (from 29.2 to 5.7 ng mL^–1^) of the IC_50_ value for paclitaxel. This was referred as the reversal index. The effect was weaker for the MCF-7/ADR cells, where a reversal index of 2.8 was obtained. It is exciting to find that the paclitaxel-loaded mPEG–SA–rapamycin micelle is far more cytotoxic towards the MCF-7/ADR cells than free paclitaxel: its IC_50_ value was 20.2 fold lower than that of free drug (Table 1). This suggests that the paclitaxel-loaded mPEG–SA–rapamycin micelles can overcome the paclitaxel resistance of MCF-7/ADR cells, allowing for effective treatment of MDR cancer at the cellular level. This result is fully consistent with our earlier observation that the amount of R6G successfully accumulated inside the MCF-7/ADR cells resulting from the R6G-micelle treatment was 14 fold as high as that treated with the free R6G.

**Table 1:** IC_50_ values of different paclitaxel formulations against the MCF-7 and MCF-7/ADR cells

Formulation	IC_50_ of PTX (μg mL^–1^)		Resistance index^*a*^	Reversal index^*b*^
	MCF-7/ADR	MCF-7		
Paclitaxel	6.2	0.0292	212	
Rapamycin–paclitaxel (3 : 1, w/w mixture)	2.2	0.0057		2.8
Paclitaxel–PEG–SA–rapamycin micelles	0.307	0.0075		20.2

^*a*^Resistance index: ratio of IC_50_ against MCF-7 over the MCF-7/ADR cells.

^*b*^Reversal index: ratio of IC_50_ for free paclitaxel to that of paclitaxel with reversal agents against the MCF-7/ADR cells.

In summary, we have shown that PEGylation of rapamycin into a pH-sensitive mPEG–SA–rapamycin not only greatly improved its water-solubility but also generated amphiphilicity, allowing for convenient micelle assembly and encapsulation of paclitaxel, a potent hydrophobic anticancer drug. The paclitaxel-loaded mPEG–SA–rapamycin nanosized micelles are stable, water-soluble, having high drug loading and capable of producing efficient, pH-triggered simultaneous dual-drug release, and hence provide an ideal solution for intracellular dual-drug co-delivery to maximise efficacy. The paclitaxel-loaded micelles exhibited ∼20 fold higher potency against the multidrug resistant MCF-7/ADR breast cancer cells over free paclitaxel. Extending this system to other drugs may establish a new general and effective strategy for dual-/multi-drug combinational therapies, allowing for effective treatment of the challenging MDR cancer tumours.
