# Supplementary material for: Self-assembled micelles of amphiphilic PEGylated rapamycin for loading paclitaxel and resisting multidrug resistant cancer cells
Source: J Mater Chem B. 2015 Jan 27;3(7):1204–7. doi: 10.1039/c4tb01633e (PMC4324122; doi:10.1039/c4tb01633e)
Supplement: Supplementary file 1 [file TB-003-C4TB01633E-s001.pdf]

This journal is © The Royal Society of Chemistry 2015

Electronic Supporting Information (ESI)

Self-assembled micelles of amphiphilicPEGylatedrapamycin for loading paclitaxel and resisting multidrug resistant cancer cells

Wei Tian[a&], JieyingLiu[a&], Yuan Guo[b], YuanyuanShen[a], Dejian Zhou*[b] and Shengrong Guo*[a,b]

[a]*School of Pharmacy, ShanghaiJiao Tong University, 800 Dongchuan Road, Shanghai200240, China.*
[b]*School of Chemistry and Astbury Centre for Structural Molecular Biology, University of Leeds, Leeds LS2 9JT, UK.*
&:equalcontributors
*:corresponding to Dr.Zhou (d.zhou@leeds.ac.uk) or Dr. S.Guo (s.guo@leeds.ac.uk or srguo@sjtu.edu.cn)

Experimental Section

1. Materials

Monomethoxypoly(ethylene glycol) (mPEG, Mn = 2000) was purchased from Fluka, USA. Rapamycin was purchased from Sigma-Aldrich (St. Louis, MO, USA). Paclitaxel was purchased from Yunnan Ziyun Biotechnology Co., Ltd. (China)and its chemical structure is shown in Scheme S1.Pyrene was purchased from AlfaAesar (USA).Penicillin, streptomycin, fetal bovine serum (FBS), and 0.25% (w/v) trypsin- 0.03% (w/v) EDTA solution were purchased from Solarbio Science & Technology Co., Ltd.(Beijing, China). RPMI-1640 medium was purchased from Gibco BRL (Gaithersberg, MD, USA).Propidium iodide (PI) was purchased from US Biological (MA, USA). All other chemicals were of analytical or HPLC grade commercially available.

2. Preparation of mPEG-SA-rapamycinconjugate

2.1 Preparation of mPEG-SAconjugate

mPEG-SAconjugate was prepared as described previously.[1] Briefly, 2.0 g of mPEG(Mn=2000, 1.0 mmol) reacted with 110mg of succinic anhydride (1.0 mmol) in the presence of 30 mg ofp-toluene

sulfonic acid (0.17 mmol). The reaction was performed under nitrogen atmosphere at 80℃ for 3 h. The resulting product was dissolved in 5 mL CH_2Cl_2, filtered, then precipitated by adding 50 mL anhydrous diethyl ether, and finally dried under vacuum to obtain 1.56gmPEG-SA(yield, 74.3%). ^1H NMR (400 MHz, $CDCl_3$, δ ppm):3.5-3.8 (m, ~176H, PEGchain -O-C**H₂**C**H₂**O-), 3.37(3H, s,C**H₃**O-), 2.65(4H, m, -O₂C-C**H₂**-C**H₂**-CO₂H).

2.2 Preparation of mPEG-SA-rapamycinconjugate

mPEG-SA-rapamycin was prepared according to the previous report[2] with a minor modification. Briefly, mPEG-SA(88 mg, 0.042 mmol)was dissolved in 10 mL of anhydrous CH_2Cl_2, and then cooled down to 0°C.107mg rapamycin (0.12 mmol),30mg DCC (0.15 mmol) and 15mg DMAP were added into it. The resulting solution was stirred at 0°Cfor 1 h, then allowing to warm up to room temperature and keep stirringfor 72h. The reaction mixture was filtered and precipitated by anhydrous diethyl ether for two times. The precipitate was dried under vacuumto get 81.1mgmPEG-SA-rapamycin (yield,64.5%). ^1H NMR (400 MHz, $CDCl_3$, δ ppm) spectra of rapamycin, mPEG-SA and mPEG-SA-rapamycin and the representative O-Me proton assignments according to ref. 10 are shown in **Figure** S1A. Thin layer chromatography (TLC, silica gel) analysis of the mPEG-SA-rapamycinconjugate yielded a single peak (silica gel, 10% MeOH in CH_2Cl_2) with a R_f value of 0.25, which is completely different from that of the mPEG-SA (**1,** R_f = 0.10), and rapamycin (**2,** R_f = 0.60), confirming that the mPEG-SA-rapamycinconjugate is pure (**Figure S1B**)

3. Water solubility measurement

The water solubility of mPEG-SA-rapamycinwas estimated by simple visual determination.[3]Briefly, a predetermined amount of mPEG-SA-rapamycinor rapamycinsample and 1 mL water were added into a microcentrifuge tube. The tube was placed in a shaking water bath at 25°Cwith a shaking speed of 100 rpm. After 24 h, the tubes were observed by the naked eyes.

4. Preparation ofmPEG-SA-rapamycinmicelles

mPEG-SA-rapamycinmicelles were prepared by a solid dispersion method.[4]Briefly, mPEG-SA-rapamycin(1 mg)wasdissolved in 200 μl acetonitrile to form a mPEG-SA-rapamycin acetonitrile solution. The solution was evaporated under reduced pressureat 25 °Cto remove acetonitrile, and then vortexed for 5 min after adding 4 mLwater.The aqueous solution wasfiltered

through 0.45μmfilter (Agela Technologies Inc., China) to produce a transparent micelles solution, which was freeze-dried and stored at 4 °C for further use.

5. Characterization of mPEG-SA-rapamycinmicelles

5.1 Critical micelle concentration (CMC) determination

The CMC of mPEG-SA-rapamycinmicelles was determined by a steady-state pyrenefluorescence methodusing a Hitachi F-7000 spectrophotometer.[5]The emission wavelength was set at 395 nm and the excitation spectra were recorded from 300 to 350 nm with a bandwidth of 5 nm. The intensity ratio at 337 and 334 nm(I_{337}/I_{334}) was used to determine the CMC.

5.2 Particle size and zetapotential(ζ) measurements

The zeta potential, particle size and size distribution of mPEG-SA-rapamycin micelles were determined by dynamic light scattering (DLS) using a ZetasizerNano ZS (Malvern Instrument) at 25 ◦C. Data are displayed as the mean value of at least three measurements ±standard deviation.

6. Preparation and characterization ofpaclitaxel-loaded mPEG-SA-rapamycinmicelles

6.1 Preparationofpaclitaxel-loaded mPEG-SA-rapamycinmicelles

Paclitaxelloaded mPEG-SA-rapamycinmicelleswere prepared by a solid dispersion method.[6]Briefly, paclitaxel (0.9mg) and mPEG-SA-rapamycin (9mg) were dissolved in 100μLacetonitrile to form a paclitaxel and mPEG-SA-rapamycin solution. The solution was evaporated under reduced pressureat 25 °Cto remove acetonitrile, and then vortexed for 5 min after adding 10 mLwater.The aqueous solution wasfiltered through a 0.45μm filter (Agela Technologies Inc., China) to produce a transparent micellar solution, which was freeze-dried and stored at 4 °C for further use.

6.2 Physico-chemical characterization of paclitaxel-loaded mPEG-SA-rapamycinmicelles

The measurements of particle size and size distribution, zeta potential of paclitaxel-loaded mPEG-SA-rapamycinmicelleswere the same as those of mPEG-SA-rapamycinmicelles, described above.

Quantification of encapsulation efficiency and drug loading.The paclitaxel-loaded mPEG-SA-rapamycinmicelles was treated with acetonitrile to get free paclitaxel solution, then

filtered with a 0.45μm syringe filter and quantitatively analyzed using high-performance liquid chromatography (HPLC) equipped with a LC-10AVP pump, a SPD-10AVP UV–vis detector (Shimadzu Co., Japan). Each sample solution (30μl) was injected at least three times into a Diamonsil C18 reversed phase column (150×4.60 mm, 5μm) preceded by a C18 guard column (Dikma, China). The mobile phase was a mixture of water and acetonitrile (50:50 v/v). The elution rate was 1.0 mL/min and the paclitaxel detection wavelength was set at 227 nm. The encapsulation efficiency and drug loading content were calculated as below [7]:

$$\text{Encapsulation efficiency}(\%) = \frac{\text{weight of paclitaxel in micelles}}{\text{weight of feeding paclitaxel}} \times 100\%$$

$$\text{Drug loading content}(\%) = \frac{\text{weight of paclitaxel in micelles}}{\text{weight of micelles}} \times 100\%$$

***In vitro* release test**.In order to create pseudo-sink condition, the *in vitro*drug release from micelles was determined in 0.01 M PBS(pH 5.0, 7.4) containing1% w/v Tween-80 [8]. 1 mL of the paclitaxel-loaded micelles was filled into a dialysis tube (molecular weight cutoff = 1,000, Snakeskin, Pierce, USA). The dialysis tube was immersed fully in 5mL of the release medium and placed in a shaking water bath at 37°C with a shaking speed of 100 rpm. At predetermined time intervals, the release medium was completely withdrawn and replaced with fresh release medium. The collected samples were diluted with acetonitrile for HPLC analysis. All measurements were determined in triplicates.

7. Cell culture

Cell culture experiments were performed in one medium (a RPMI-1640 medium with 10% fetal bovine serum (FBS) and 1% penicillin-streptomycin),at 37°C under 5% CO_2 atmosphere in an incubator. MCF-7 cells were obtained from Shanghai Institute of Cell Biology, Chinese Academy of Sciences

8. Cell cycle analysis

MCF-7 cells were seeded at a density of 1.5×105 cells/mL (2 mL medium/well) in 6-well plates and incubated inmediafor 24 h and then treated with rapamycin and mPEG-SA-rapamycin at an equivalent rapamycinconcentration of 20 ng/mL for 24 h at 37 °C. After removal of the media and washing with cold PBS three times, the cells were collected by centrifugation and then fixed with 70% pre-cooled alcohol overnight at 4 °C. Afterwards, the cells were washed to eliminate alcohol

and then treated with RNase A (0.1 mg/mL) and PI (0.1 mg/mL) in the dark for 30 min at 37 °C, the cell cycle distribution was analyzed by flow cytometry (FACS Calibur, BD, USA).

9. *In vitro*cytotoxicity evaluation

The *in vitro*cytotoxicity assays were performed. Briefly, MCF-7 cells in their logarithmic growth regime were seeded and incubated in 96-well plates at a density of 5000 cells/well in 200 μL medium for 24 h, then, the culture medium in each well was carefully replaced by 200 μL of fresh medium containing various concentrations of mPEG-SA-rapamycin, the paclitaxel-loaded mPEG-SA-rapamycinmicelles, paclitaxel+rapamycin (1 : 3, w/w), free paclitaxel+mPEG-SA-rapamycin (paclitaxel : equivalent rapamycin, 1 : 3, w/w), free paclitaxelor rapamycin, respectively. After 72 h incubation and removal of the media, each well was added with 180 μL fresh medium and 20 μL of PBS containing 5 mg/mL MTT. The media were discarded after 4 h incubation, and 200 μL of DMSO was added into each well to dissolve the formed formazan crystals. The absorbance at 570 nm was recorded on an ELISA plate reader (Varioskan Flash). Cell viability of untreated cells is defined as 100%. Each experiment was done with ten parallel samples. Cells incubated in medium containing 0.1% DMSO were also as controls.

10. Cellular uptake study

Cells were seeded at a density of 5 × 10^5 cells/mL (2 mL medium/well) in 12-well plates and allowed to adhere to the plates for 24 h. After removal of medium, 2 mL of the fresh medium with 0.25 μM(equivalent Rhodamine 6G) of Rhodamine 6G or the Rhodamine 6G-loaded mPEG-SA-rapamycin micelles was added into each wells. At the designated time points(2,4,8 and 24h), the cells were washed three times with ice-cold PBS, collected by centrifugation and the resuspended in 0.5 mL PBS. The amount of uptake of Rhodamine 6G was measured on a flow cytometer (BD LSRFortessa, Becton Dickinson) using a 508 nm argon laser for excitation and a 560 nm emission band-pass filter to detect the fluorescence ofRhodamine 6G.

11. Cellular efflux study

Cells were seeded at a density of 5 × 10^5 cells/mL (2 mL medium/well) in 12-well plates and allowed to adhere to the plates for 24 h. After removal of medium, 2 mL of the fresh medium with 0.25 μM(equivalent Rhodamine 6G) of Rhodamine 6G or the Rhodamine 6G-loaded mPEG-SA-rapamycin micelles was added into each wells and incubated for 4h. Afterwards, the cells were washed three times with ice-cold PBS and incubated in fresh medium. At predetermined time intervals, The amount of Rhodamine 6G retained within the MCF-7 or MCF-7/ADR cellswas measured on a flow cytometer (BD LSRFortessa, Becton Dickinson) using a 508 nm argon laser for excitation and a 560 nm emission band-pass filter to detect the fluorescence ofRhodamine 6G.

References:

[1] F.Xia, Y. J.Yuan and J. C.Wu,*Bioorg. Med. Chem. Lett.*, 2002, 12, 3301-3303.

[2] A. Battaglia, A. Guerrini, E. Baldelli, G. Fontana, G. Varchi, C. Samori and E. Bombardelli, *Tetrahedron Lett.*2006; 47: 2667-2670.

[3] L. Song, Y. Shen, J. Hou, L. Lei, S. Guo and C. Qian.*Coll. Surf. A*, 2011, 390, 25-32.

[4] S. C. Kim, D.W. Kim, Y.H. Shim, J.S. Bang, H.S. Oh, S.W. Kim and M.H. Seo, *J. Control. Release* 2001; 72: 191–202.

[5] H. Wu , L. Zhu and V. P. Torchilin. *Biomaterials* 2013, 34, 1213–1222.

[6] C. Wang, Y. J. Wang, Y. J. Wang, M. Fan, F. Luo, Z. Y. Qian. *Int. J. Pharm*. 2011, 414: 251– 259.

[7] Z. Wei , J. Hao, S. Yuan, Y. Li, W. Juan, X. Sha and X. Fang. *Int. J. Pharm*. 2009, 376: 176–185.

[8] Y. Wang, C. Wang, C. Gong, Y. Wang, G. Guo, F. Luo and Z. Qian. *Int. J. Pharm*. 2012, 434, 1– 8.

[9] A. W. G. Alani, Y. Bae, D. A. Rao and G. S. Kwon.*Biomaterials*, 2010, 31, 1765–1772.

[10] J. A. Findlay and L.Adics.*Can. J. Chem*.1980, 58,579.

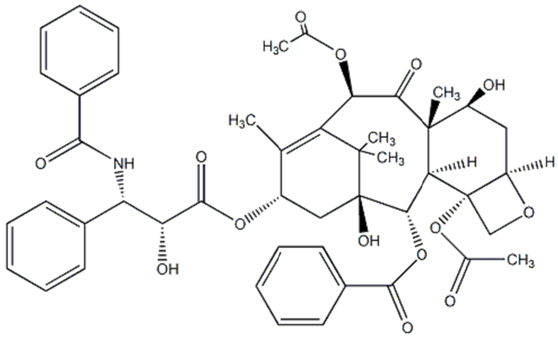

Scheme S1. Chemical structure of paclitaxel

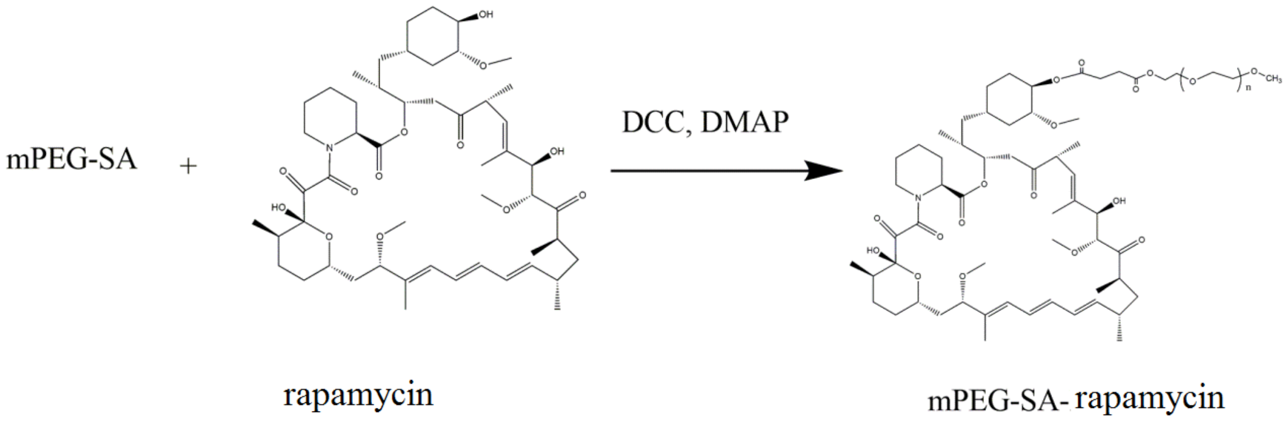

Scheme S2. The synthetic route to the mPEG-SA-rapamycinconjugate.

Table S1.Cell cycle distributions of MCF-7 cells after incubation with rapamycin ormPEG-SA-rapamycin (n = 3)

	G_0/G_1 (%)	S(%)	G_2/M(%)
Control	56.79	28.63	14.58
Rapamycin	69.42	19.19	11.39
mPEG-SA-rapamycin	68.14	21.30	10.56

Table S2. Cytotoxicity of free rapamycin,mPEG-SA-rapamycinagainst MCF-7 or MCF-7/ADR cells

	IC_{50} of equivalent rapamycin (μg/mL)		Resistance index
	MCF-7/ADR	MCF-7	
Rapamycin	10.50	0.20	52.5
mPEG-SA-rapamycin	3.48	1.02	3.4

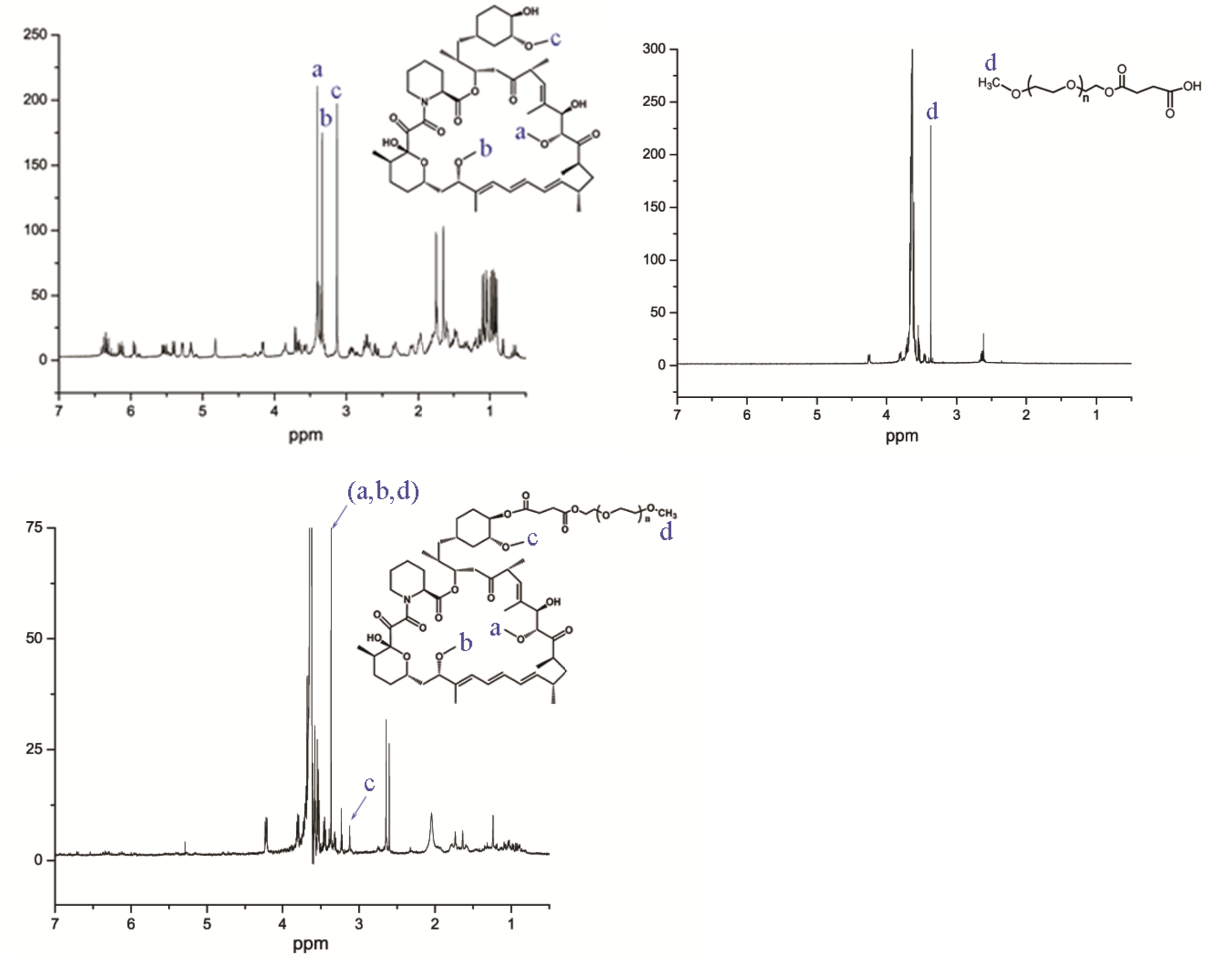

Figure S1A: ^{1}H NMR spectra of rapamycin, mPEG-SA and mPEG-SA-rapamycin in $CDCl_3$ (400 MHz, δ ppm). Representative assignments of the O-Me groups in the each compound are given in each figure. The integrated peak area ratio of proton **c**: (**a** + **b** + **d**) in the mPEG-SA-rapamycin spectrum is found to be 1:3.04, consistent with the expected proton ratio. The integrated proton area ratios broadly agree with what is expected from a 1:1 conjugate.

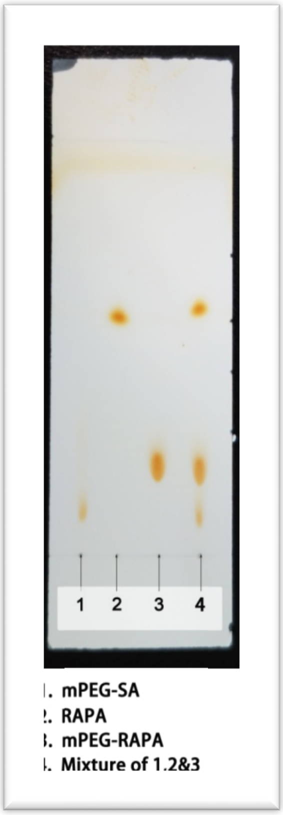

Figure S1B: TLC analysis photograph (silica gel, 10% MeOH in CH_2Cl_2) of the mPEG-SA (**1**), rapamycin (**2**), mPEG-rapamycin conjugate (**3**) and a mixture of mPEG-SA, rapamycin, and mPEG-rapamycin conjugate (**4**) after iodine stain. It is clear that the mPEG-rapamycin conjugate is pure, contianing just a single specie.

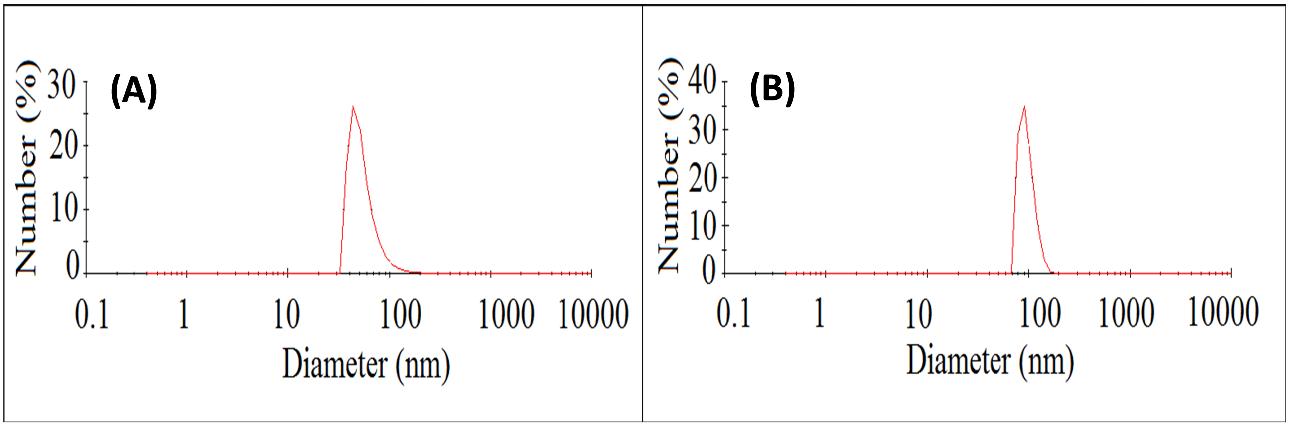

Figure S2. Hydrodynamic diameter distributions of the mPEG-SA-rapamycin micelles (**A**) and the paclitaxel loaded mPEG-SA-rapamycin micelles (**B**) measured by dynamic light scattering.

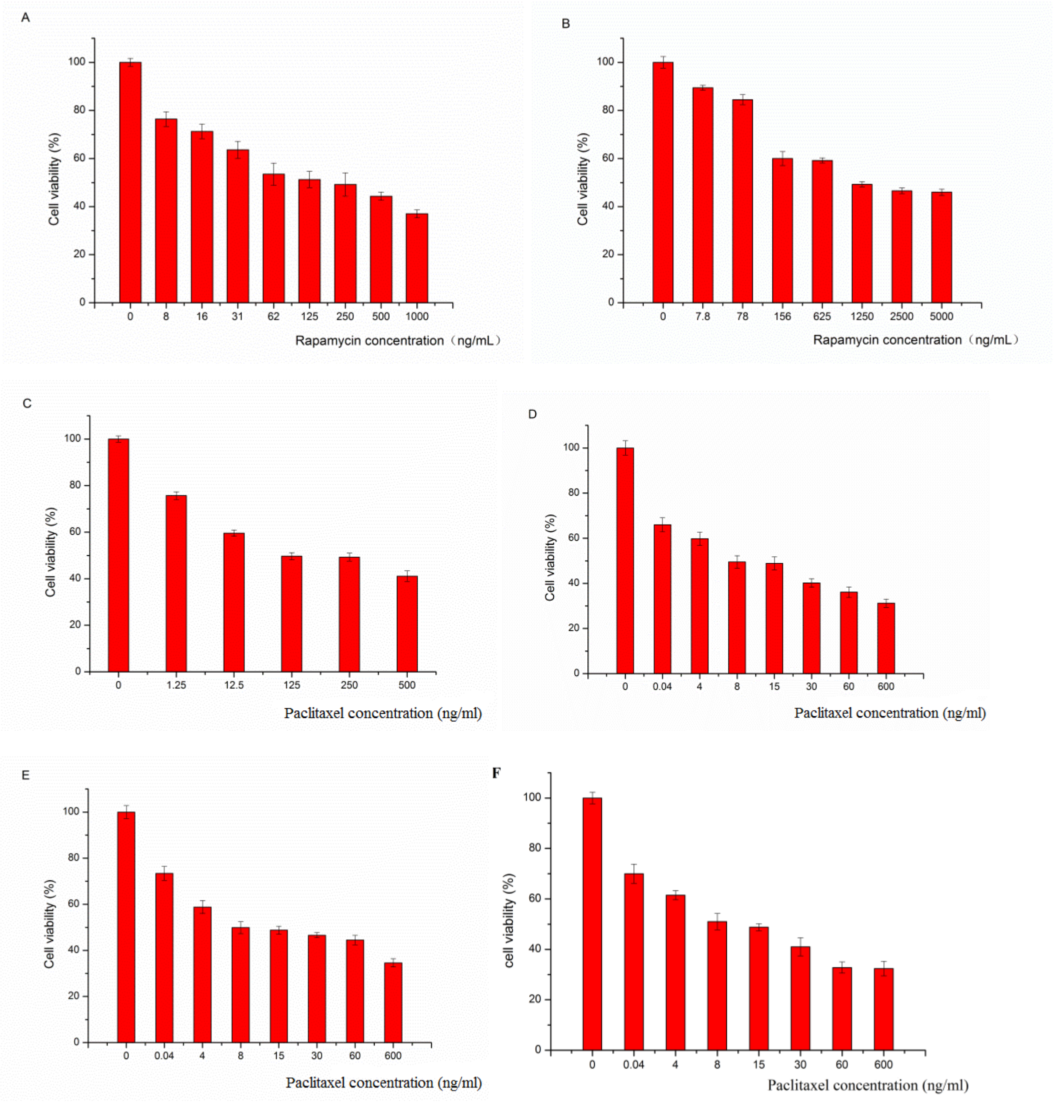

Figure S3. Dose-dependent *in vitro*cytotoxicitiesof different drug formulations against the drug-sensitive MCF-7 cell line. (**A**) rapamycin, (**B**) mPEG-SA-rapamycin, (**C**) paclitaxel, (**D**) rapamycin+paclitaxelphysical mixture (3:1 w/w), (**E**) paclitaxel+mPEG-SA-rapamycinphysical mixture (3:1, w/wequivalent rapamycin : paclitaxel) and (**F**) the paclitaxel-loaded mPEG-SA-rapamycinmicelles. Data are presented asmean ± SD (n=5).

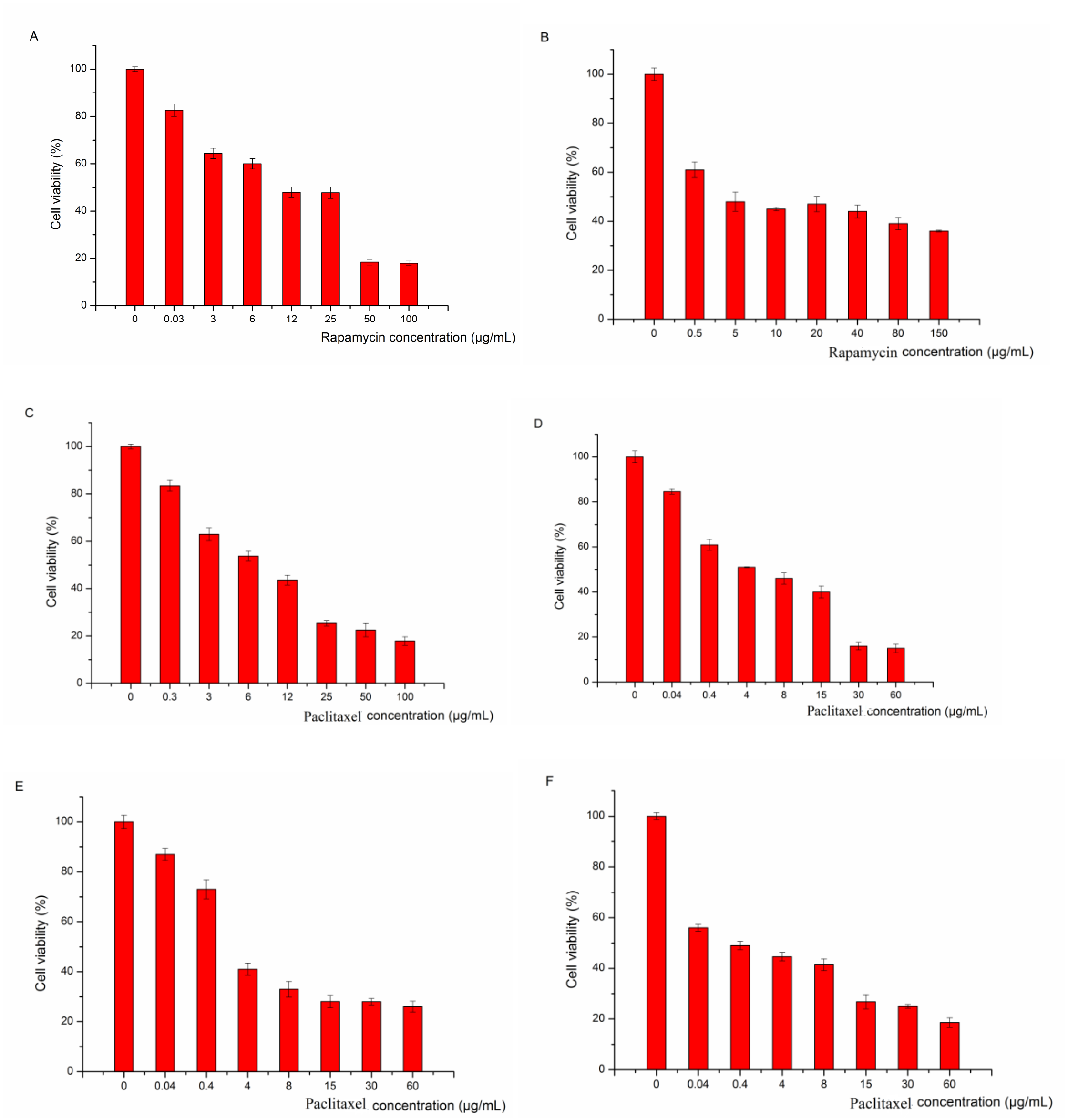

Figure S4.Does-dependent *in vitro*cytotoxicities of different drug formulationsagainst the multidrug resistant MCF-7/ADR cell line.(**A**) rapamycin, (**B**) mPEG-SA-rapamycin, (**C**) paclitaxel, (**D**) rapamycin+ paclitaxelphysical mixture (3:1, w/w), (**E**) free paclitaxel+ free mPEG-SA-rapamycinphysical mixture (3:1 w/w equivalent rapamycin:paclitaxel) and (**F**) the paclitaxel-loaded mPEG-SA-rapamycinmicelles. Data are presented asmean ± SD (n=5).
